# Activation of AMPA Receptors in the Lateral Habenula Produces Anxiolytic Effects in a Rat Model of Parkinson’s Disease

**DOI:** 10.3389/fphar.2022.821975

**Published:** 2022-01-25

**Authors:** Jin Zhang, Xiaobing Wang, Rick E. Bernardi, Jun Ju, Shoupeng Wei, Zhiting Gong

**Affiliations:** ^1^ State Key Laboratory of Chemical Oncogenomics, Guangdong Provincial Key Laboratory of Chemical Genomics, Shenzhen Graduate School, Peking University, Shenzhen, China; ^2^ Department of Anatomy, College of Preclinical Medicine, Dali University, Dali, China; ^3^ Institute of Psychopharmacology, Central Institute of Mental Health, Medical Faculty Mannheim, Heidelberg, Germany; ^4^ Brain Research Centre and Department of Biology, Southern University of Science and Technology, Shenzhen, China; ^5^ Tomas Lindahl Nobel Laureate Laboratory, Precision Medicine Research Centre, Seventh Affiliated Hospital, Sun Yat-sen University, Shenzhen, China

**Keywords:** Parkinson’s disease, AMPA receptors, lateral habenula, anxiety, serotonin, dopamine

## Abstract

**Background:** Parkinson’s disease (PD) is commonly accompanied with anxiety disorder, however, the mechanisms underlying PD-mediated anxiety remain elusive. The lateral habenula (LHb) is a critical brain region that influences the activity of the monoaminergic system in the midbrain and consequently modulates anxiety. Most neurons in the LHb express AMPA receptors (AMPARs). The PD model for the pharmacological intervention of AMPA receptors was established by the unilateral lesion of the substantia nigra pars compacta (SNc) with 6-hydroxydopamine (6-OHDA).

**Methods:** The AMPAR agonist (*S*)-AMPA and antagonist NBQX were microinjected into the LHb, respectively, to examine whether anxiety-like behaviors were altered in sham-operated and SNc-lesion rats, measured with the paradigms of the open-field test (OPT) and elevated plus maze (EPM). Furthermore, dopamine (DA) and 5-hydroxytryptamine (5-HT) levels in the basolateral amygdala (BLA) were measured using *in vivo* microdialysis immediately following the injections of (*S*)-AMPA and NBQX into the LHb.

**Results:** Activation of LHb AMPA receptors by (*S*)-AMPA produced anxiolytic-like behaviors and enhanced the extracellular DA and 5-HT in the BLA. Conversely, NBQX induced anxiety-like effects and suppressed the extracellular DA and 5-HT in the BLA. In addition, the minimal doses inducing the effects in the SNc-lesion rats were lower than those in sham-operated rats.

**Conclusion:** These findings suggest that the effects of AMPA receptors in the LHb on anxiety-like behaviors likely involve the extracellular levels of DA and 5-HT in the BLA. The present results may improve our understanding of the neuropathology and/or treatment of PD.

## Introduction

The lateral habenula (LHb) is a crucial structure of the epithalamus, and neuronal axons from the LHb project onto monoaminergic neurons in the midbrain (Bernard and Veh, 2012). Increasing studies suggest that the LHb is highly involved in the regulation of anxiety-like behaviors; for example, the rats refraining from chronic excessive alcohol consumption showed an anxiety-like phenotype, an increase in glutamate release, and hyperactivity of LHb neurons (Kurumaji et al., 2003; Berger et al., 2018). Moreover, blocking glutamate transmission or LHb neuronal activity rescued anxiety-like behaviors in alcohol-depriving rats and suppressed alcohol intake upon the reexposure to alcohol (Li et al., 2017; Shah et al., 2017; Kang et al., 2018; Fu et al., 2020). In addition, the LHb also plays an important role in chronic nicotine-induced anxiety (Casarrubea et al., 2021) and bilateral electrolytic lesion of the LHb may induced an anxiolytic-like effect (Pobbe and Zangrossi, 2008; Casarrubea et al., 2015). The amygdaloid complex is a heterogeneous cluster of 13 nuclei in the medial temporal lobe. Its nuclei are divided into three groups based on the anatomical structure, one of the groups includes the lateral and basal nuclei [oftentimes referred to together as the basolateral nucleus (BLA)], as well as the accessory basal nucleus. Increasing evidence indicates that the BLA is closely related to anxiety-like behaviors, because the levels of extracellular dopamine (DA) and serotonin (5-HT) were increased in the BLA in the anxiety-like state (Sah et al., 2003; Sun et al., 2018; Liu et al., 2019; Du et al., 2021).

Parkinson’s disease (PD) is a neurodegenerative disease with a high incidence rate among elderly people, closely associated with a severe loss of dopaminergic neurons in substantia nigra. PD is traditionally defined by motor features such as: resting tremor, bradykinesia and rigidity. However, the non-motor symptoms, such as anxiety, can also dramatically affect a patients’ quality of life; approximately 25–49% of PD patients are affected by anxiety (Pontone et al., 2009). Previous studies have demonstrated that degenerated neurotransmitter systems in the cerebral cortex and brainstem in PD may represent an underlying cause of anxiety (Walsh and Bennett, 2001; Bassetti, 2011; Prediger et al., 2019), though the possible neurologic explanations of anxiety in PD remain complicated and poorly understood (Hurtado-Lorenzo et al., 2004; Bassetti, 2011). Furthermore, many tests have shown that 6-hydroxydopamine (6-OHDA) lesions of the nigrostriatal circuit induce anxiety-like responses as measured by different paradigms of anxiety evaluation (Tadaiesky et al., 2008; Li et al., 2012; Hui et al., 2015; Sun et al., 2015; Sun et al., 2018) and increase the firing rates of LHb neurons (Wang et al., 2017; Zhang et al., 2019). These results supported the essential role of LHb in PD-associated anxiety.

Most neurons located in the LHb are glutamatergic (Brinschwitz et al., 2010; Li et al., 2011; Aizawa et al., 2012) and express α-amino-3-hydroxy-5-methyl-4-isoxazole-propionic acid receptors (AMPARs) (Li et al., 2011; Meye et al., 2013; Shabel et al., 2014). AMPARs are built by four subunits (GluR1–4) and categorized into GluR2-lacking (Ca^2+^-permeable AMPA receptors, CP-AMPARs) and GluR2-containing AMPARs (Ca^2+^-impermeable AMPA receptors, CI-AMPARs) (Greger et al., 2017); in other words, the lack or presence of the GluR2 subunit renders AMPA receptors permeable (CP-AMPARs) or impermeable (CI-AMPARs), respectively, to calcium (Cull-Candy et al., 2006). The LHb receives strong excitatory inputs, including the Globus Pallidus Internal Segment/Entopeduncular Nucleus (GPi/EPN), the lateral hypothalamus (LHA) and lateral preoptic area (LPO) which exerting excitatory effects on the targeted LHb cells (Hu et al., 2020). Further, several studies from our laboratory have found AMPARs in the LHb effected the synthesis and release of DA and 5-HT in depression. Anxiety disorders may precede or be accompanied by depression and, in such cases, even when the depression is treated, anxiety may remain (Butala et al., 2019). However, little has been known about the role of the AMPARs in the LHb on the regulation of anxiety-like behaviors in Parkinsonian animals up to now. Thus, the aim of the present study was to investigate the effects of activating and blocking LHb AMPARs on anxiety-like behaviors and changes of dopamine (DA) and serotonin (5-HT) release in the ipsilateral basolateral nucleus of BLA.

## Materials and Methods

### Animals and Drugs

Male Sprague-Dawley rats (weighing 280–330 g, provided by the Experimental Animal Center of Xi’an Jiaotong University, Xi’an, China) were housed in a room with constant temperature (21 ± 1°C) under a regular light/dark cycle schedule (light on, 8:00–20:00 h). They had *ad libitum* access to standard food and drinking water. All the experiments were strictly conducted in accordance with the *National Institutes of Health Guide for Care and Use of Laboratory Animals* (NIH publication, 8th edition, 2011), and All the experimental procedures were strictly abided by the National Institute of Health Guidelines and approved by the Animal Care and Use Committee of the Xi’an Jiao Tong University. All efforts were made to minimize the number of animals used and reduce their discomfort.

Desipramine hydrochloride, 6-OHDA hydrochloride, apomorphine (Sigma-Aldrich, USA), (*S*)-α-amino-3-hydroxy-5-methyl-4-isoxazolepropionic acid [(*S*)-AMPA, a selective AMPARs agonist] and 2,3-dioxo-6-nitro-1,2,3,4-tetrahydrobenzo[f]quinoxaline-7-sulfonamide disodium salt (NBQX, a potent AMPARs antagonist) (Tocris, UK) were utilized in this study. Desipramine, (*S*)-AMPA and NBQX were dissolved in saline, while 6-OHDA and apomorphine were prepared in saline containing 0.02% ascorbic acid. All injections of the drugs were freshly prepared just prior to use.

### 6-OHDA Lesion Surgeries and 6-OHDA Model Validation

**FIGURE 1:**
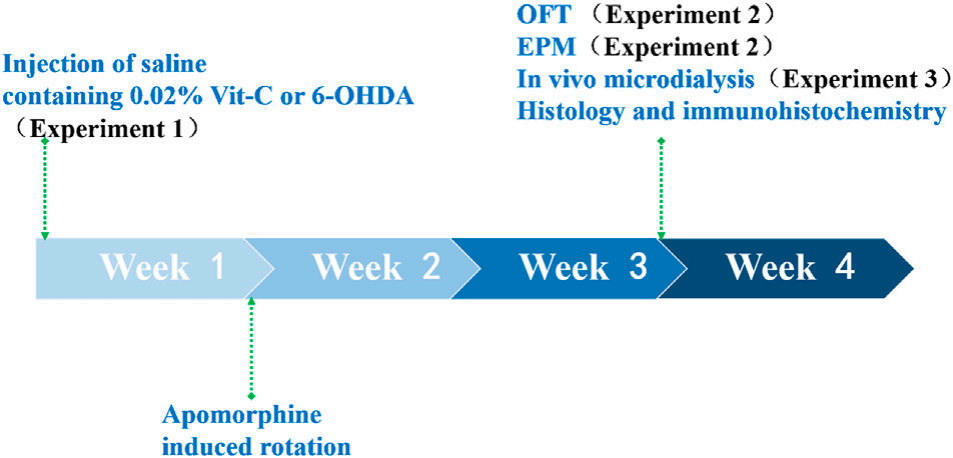
We first validated the effectiveness of the PD rat model. SNc-lesion (*n* = 23) and sham-operated (*n* = 24) rats were used to demonstrate that 6-OHDA microinfusions into the SNc and VTA caused a significant reduction in the number of TH-ir neurons in both brain areas using immunohistochemistry. First, the brain sections were blocked with 3% bovine serum albumin (BSA) in PBS containing 0.3% Triton X-100 at room temperature for 0.5 h and then incubated with anti-TH monoclonal antibodies (1:800, Chemicon, CA, USA) at 4°C for 48 h. Next, the brain sections were incubated with biotinylated anti-mouse IgG (1:200, Chemicon) for 2 h and then with avidin–biotin–peroxidase complex (1:100, Vector Laboratories, CA, USA) for 2 h at room temperature. Finally, they were exposed to a solution of 0.05% 3,3′-diaminobenzidine (Sigma-Aldrich) containing 0.01% H_2_O_2_ for 10–15 min at room temperature, serving as chromogen in the subsequent visualization reaction. After TH staining, the brain sections were rinsed, mounted onto gelatin-coated slides, dehydrated, cleared in xylene and coverslipped. Then counting the dopaminergic neuron bodies in the SNc and VTA was performed within the representative two sections per animal. A neuron was considered when intact, round with clear nucleus and/or cytoplasm. The full extent of the structure in each section was investigated in both the lesioned and non-lesioned hemisphere. Only the sections where the lateral and medial parts of the VTA and SNc were clearly divided by the medial terminal nucleus of the accessory optic tract level were chosen for the further analyses of tyrosine hydroxylase immunoreactive (TH-ir) neurons. Only the rats with a total or subtotal loss of TH immunoreactivity within the SNc were taken for analyzing electrophysiological recordings (Wang et al., 2009b).

After being anesthetized with sodium pentobarbital (40 mg/kg, i.p.), rats were fixed in a customized stereotaxic frame (SN-2N; Narishige, Tokyo, Japan) and administered an infusion of 6-OHDA (8 μg/4 μl) or saline containing 0.02% ascorbic acid (sham-operated) into the SNc of the left hemisphere (AP–5.0, ML–2.0, DV–7.3, relative to bregma; Paxinos et al., 1980). To protect noradrenergic terminals, each rat received an injection of desipramine (25 mg/kg, i.p.) approximately 30 min before 6-OHDA administration. One week after surgery, turning behavior induced by apomorphine (0.05 mg/kg, s.c.) was monitored to assess the effectiveness of the SNc lesion, and rats exhibiting more than 20 contralateral turns per 5 min were applied into the further tests (Wang et al., 2009a). All rats used in this study demonstrated >35 turns per 5 min. All experiments were conducted in the fourth week after the lesion surgeries. Experiments are summarized in Figure 1.

A separate group of SNc-lesion (*n* = 28) and sham-operated (*n* = 28) rats were utilized to investigate the degree of the degeneration of striatal dopaminergic neurons. The brains of rats were removed immediately after they were decapitated. The striatum ipsilateral to sham or DA lesion were collected. The concentrations of DA in the striatal structure were measured by reverse-phase high performance liquid chromatography (HPLC) with electrochemical detection (ECD). Only rats with a depletion of DA (>90%) in the ipsilateral striatum were taken to analyze the data (Han et al., 2016).

### Behavioral Testing (Open Field Test and Elevated Plus Maze)

SNc-lesion (*n* = 10) and sham-operated (*n* = 10) rats were mounted with a unilateral stainless steel cannula 1 mm above the LHb (AP -3.7, ML–0.8, DV–3.7; Paxinos et al., 1980) through a hole drilled in the skull. A cannula was fixed to the skull with 3 stainless steel screws and then covered with dental acrylic cement. A dummy was inserted into the cannula to prevent potential obstruction, and rats were given a 1-week recovery period prior to behavioral tests.

SNc-lesion and sham-operated rats were administered one of the following combinations of infusions: saline/(*S*)-AMPA (doses: 0.01875, 0.0375, or 0.075 μg), saline/NBQX (0.25, 0.5, or 1.0 μg), or (*S*)-AMPA (0.075 μg)/NBQX (1.0 μg). During infusions, rats were gently handled by the experimenters, the stylet was withdrawn from the guide cannula, and a microinjector that was inserted alongside the guide cannula extended 1 mm beneath the cannula tip. The microinjector was connected to a 1-μl microsyringe through PE-10 tubing. The drugs were injected into the left LHb at a speed of 0.3 μl/min. Behavioral tests were conducted 10 min after the intra-LHb microinjection, and the interval between 2 subsequent injections was 5 min. Behavioral experiments were performed on the subsequent days, with the open field test (OFT) followed 24 h later by the elevated plus maze (EPM).

A paradigm of the OFT was designed to indicate spontaneous locomotor activity and anxiety-like behavior in rodents. The apparatus was constructed with a white Plexiglass floor of 100 cm wide × 100 cm long and 4 white walls 40 cm high. The floor area was further divided into 25 squares of 20 cm × 20 cm by black lines. Tests were performed under low-light environment (35–45 lux). Ten minutes post the microinfusion, the rat was placed in the center of the open field and allowed to freely explore the area for 5 min. The number of squares crossed (horizontal locomotion) and rearings (vertical activity) was recorded to indicate spontaneous locomotor activity, and the percentage of time spent in the central area (60 cm × 60 cm) of the open field was taken as an index of anxiety, calculated as time spent in the central area (s)/300 (s) × 100 (Tadaiesky et al., 2008; Sun et al., 2015; Sun et al., 2018; Liu et al., 2019; Du et al., 2021).

A paradigm of the EPM test was taken to evaluate spontaneous locomotor activity and anxiety-like behavior as well. The EPM apparatus had a central platform (10 cm × 10 cm) connected to two open arms (50 cm long × 10 cm wide) and two closed arms (50 cm long × 10 cm wide, walls 45 cm high) in the shape of crossing, elevated 50 cm above the floor. The illumination in the open arms, closed arms and central platform was 16, 16 and 4 lux, respectively. Twenty-4 hours following the OFT, intra-LHb infusions were repeated as above in each group. Ten minutes later, each rat was put in the center of the maze facing an open arm, while the behavior was recorded for 5 min. The number of entering the open arms was used as an indicator of spontaneous locomotor activity. The percentage of the open-arm entries [(number of open arm entries/number of open closed arm entries) × 100] and percentage of time spent in the open arms [(time in open arms/time in open closed arms) × 100] were calculated. The percentages of time spent and the entries into open arms were used to reflect the anxiety level (Tadaiesky et al., 2008; Sun et al., 2015; Sun et al., 2018; Liu et al., 2019; Du et al., 2021).

### *In Vivo* Microdialysis and Neurochemistry

SNc-lesion (*n* = 8) and sham-operated (*n* = 8) rats were stereotaxically implanted with guide cannulae into the left LHb (AP -3.7, ML–0.8, DV–3.7) for drug injection and the left BLA (AP -2.5, ML–4.9, DV–7.0; Paxinos et al., 1980) for microdialysis. A guide cannula was secured to the skull with dental acrylic cement. After a 24 h recovery period, microdialysis was conducted in unanesthetized, freely moving rats. We inserted microdialysis probes (membrane length 2 mm; Eicom, Kyoto, Japan) through the guide cannula and perfused at 1 μl/min with Ringer’s solution (147 mM NaCl, 4 mM KCl, 2.3 mM CaCl_2_).

Following a 2 h equilibration period, dialysis samples were collected every 10 min for 30 min, and the 3 fractions as the baseline values before the drug injections in the two groups of rats. To monitor the alterations in the extracellular levels of DA and 5-HT in the BLA after the intra-LHb infusion of (*S*)-AMPA (0.0375 μg) or NBQX (0.5 μg), dialysis samples were collected every 10 min for 60 min, and the concentrations were immediately measured the HPLC (Alexys Uhplc; Antec, Zoeterwoude; Netherlands) with an ECD (Decade II, Antec; HPLC-ED) as previously described (Wang et al., 2017).

### Immunohistochemistry and Histology

After all experiments were finished, rats were administered with an overdose of urethane and transcardially perfused with saline followed by 4% paraformaldehyde. The brains were quickly removed, fixed in 4% paraformaldehyde for 4 h and cryoprotected in 30% sucrose solution until saturation. Brain slices were cut at 40 μm with a cryostat microtome (Olympus, Tokyo, Japan). Brains slices from rats in Experiments 1, 2, and 3 were used for tyrosine hydroxylase (TH) immunohistochemical staining in the SNc and ventral tegmental area (VTA), as previously described (Wang et al., 2009a). Brains slices from rats in Experiments 2 and 3 were also stained with cresyl violet to verify anatomical placement of the cannulae and microdialysis probes. In the present study, behavioral and microdialysis data were only analyzed from the rats with a nearly complete loss of TH immunoreactive (TH-ir) neurons in the left SNc, and the correct anatomical placement of the cannulae, recording sites and microdialysis probes. Only rats with a depletion of DA tissue content >90% were considered for the further analysis of behavioral tests and *in vivo* microdialysis.

### Data Analysis and Statistics

All data represent as the mean ± SEM (standard error of mean). Statistical analyses were performed with SigmaStat 3.5 (Systat, San Jose, CA, USA). Data from behavioral tests and *in vivo* microdialysis data were analyzed using two-way ANOVA followed by Bonferroni’s test. Immunohistochemical data were assessed using an independent samples t-test. The significance level was set at *p* < 0.05.

## Results

### 6-OHDA Model Validation

**FIGURE 2:**
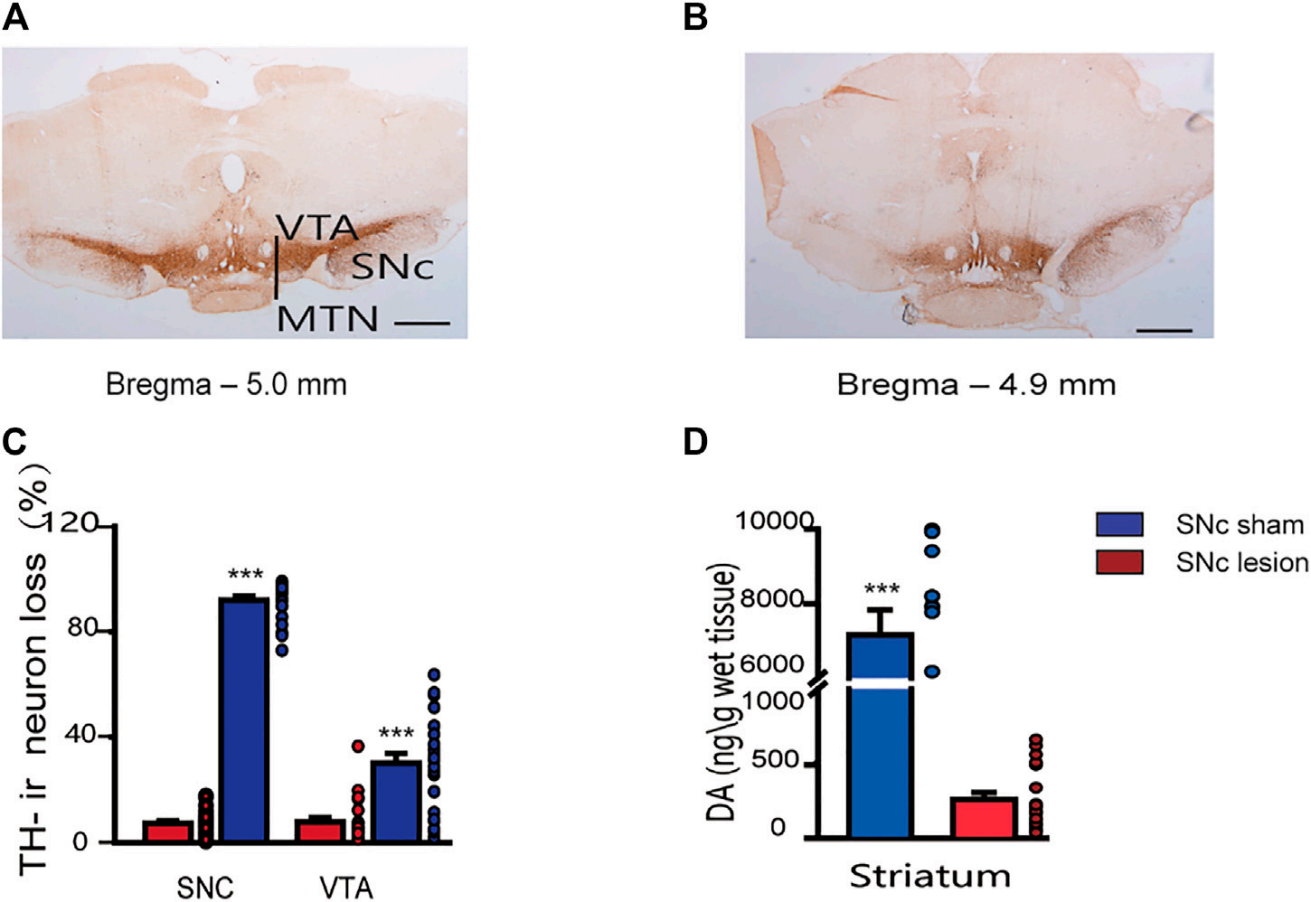
Staining of dopaminergic neurons in the SNc and VTA, and DA tissue content in the striatum in sham-operated and SNc-lesion rats. Photomicrographs show TH immunostaining of the SNc and VTA in sham-operated **(A)** and SNc-lesion **(B)** rats. A unilateral injection of 6-OHDA into the SNc in a rat led to a nearly complete loss of TH-ir neurons in the SNc and a partial loss in the VTA, compared to sham-operated rats [**(C)**; sham-operated: *n* = 24 rats/group; SNc-lesion: *n* = 23 rats/group]. A unilateral injection of 6-OHDA also decreased the DA level in the ipsilateral striatum compared to sham-operated rats [**(D)**; *n* = 28 rats/group]. ***p < 0.001 *vs*. sham-operated rats; unpaired Student’s *t*-test. Data are represented as means ± SEM. MTN, medial terminal nucleus of the accessory optic tract. Scale bars: A, B = 500 μm.

Unilateral lesions of the SNc induced an almost complete loss of TH-ir neurons in the ipsilateral SNc (-92%; *p* < 0.001) and a partial loss in the VTA (−30%; *p* < 0.001, unpaired Student’s t-test; Figure 2A–C), compared to the sham-operated rats. Lesions of the SNc also resulted in a significantly decrease in the tissue content of DA in the ipsilateral striatum (−96%; *p* < 0.001, unpaired Student’s t-test; Figure 2D).

### Effects of SNc Lesions and Pharmacological Manipulations of LHb AMPARs on Locomotor Activity in the OFT

**FIGURE 3:**
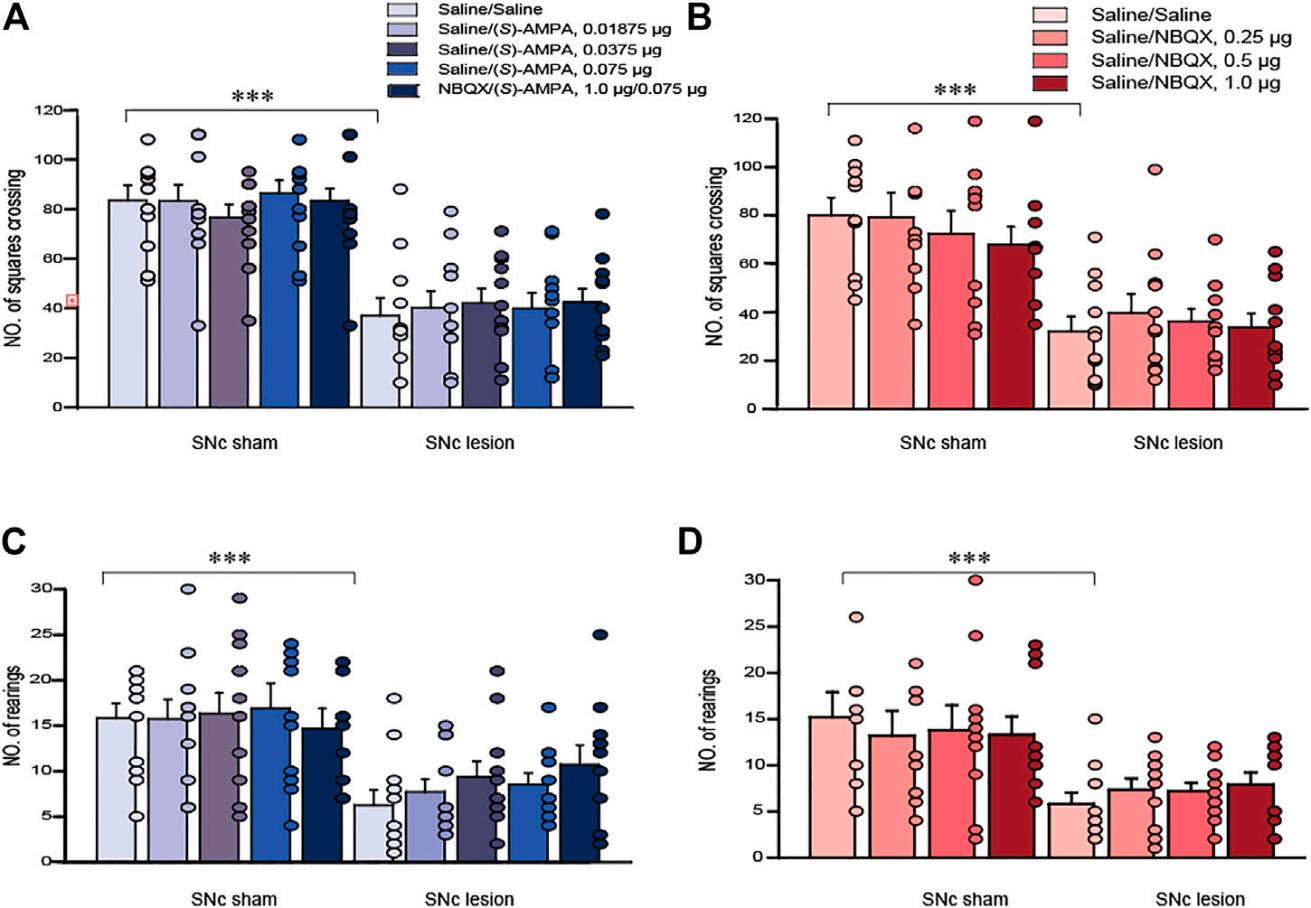
The effects of 6-OHDA lesions and the intra-LHb microinjections of (*S*)-AMPA and NBQX as the AMPARs agonist and antagonist, respectively, on spontaneous locomotor activity in the OFT. Unilateral SNc lesions decreased the number of squares crossed (A, B; horizontal movement) and rearings (C, D; vertical movement) in rats compared to that in sham-operated ones. Intra-LHb injections of (*S*)-AMPA, NBQX/(*S*)-AMPA or NBQX did not alter the number of squares crossed **(A,B)** or rearings **(C,D)** relative to the saline/saline injections in either sham-operated or 6-OHDA lesioned rats. ***p < 0.001 *vs*. sham-operated rats; two-way ANOVA followed by Bonferroni’s test. Data are represented as means ± SEM; *n* = 10–12 rats/group.

In terms of horizontal and vertical activity in rats, we investigated the effects of the unilateral lesions of the SNc and the intra-LHb microinjection of saline/saline, saline/(*S*)-AMPA, saline/NBQX or (*S*)-AMPA/NBQX in the OFT. A two-way ANOVA (lesion × drug) revealed a significant effect of lesion on locomotor activity (*F*_(1, 105)_ = 127.536, *p* < 0.001; *F*_(1, 76)_ = 56.196, *p* < 0.001 for horizontal activity, Figures 3A,B; *F*_(1, 105)_ = 33.994, *p* < 0.001; *F*_(1, 76)_ = 25.357, *p* < 0.001 for vertical activity, Figures 3C,D), but no effects of drug and no interactions. *Post hoc* analyses revealed that intra-LHb drug treatments did not affect locomotor activity relative to saline/saline treatment in either sham-operated or SNc-lesion rats.

### Effects of SNc-Lesion and Pharmacological Manipulations of LHb AMPARs on Anxiety-like Behaviors in the OFT

**FIGURE 4:**
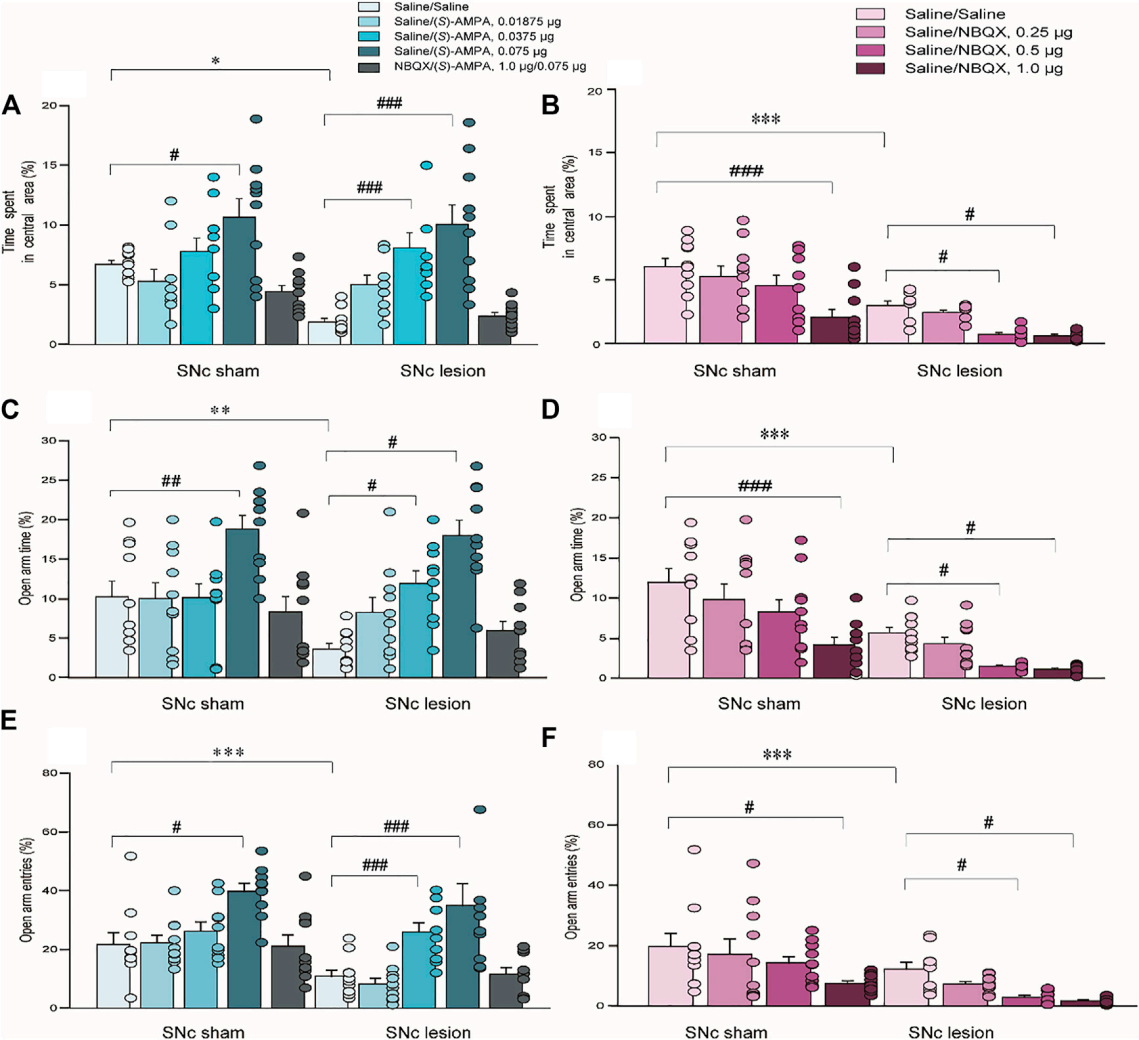
The effects of 6-OHDA lesions and the intra-LHb microinjections of (S)-AMPA and NBQX as the AMPAR agonist and antagonist, respectively, on anxiety-like behaviors measured using the OFT and EPM. Unilateral SNc lesions reduced the percent time spent in the central area in the OFT **(A,B)**, the percent time entering the open arms of the EPM **(C,D)**, and open-arm entries in the EPM **(E,F)**, compared to that in sham-operated rats. In both groups, an intra-LHb injection of (*S*)-AMPA increased the percentage of time spent in central area **(A)**, and the percent open-arm time **(C)** and open arm entries **(E)** relative to the saline/saline injection in lesioned rats; whereas the injection caused anxiolytic-like behaviors in both groups **(A,C,E)**. Pretreatment with NBQX (1.0 μg/0.3 μl) administered into the LHb blocked the effects of (*S*)-AMPA (0.075 μg/0.3 μl) on anxiety-like behaviors. Intra-LHb administration of vehicle/NBQX significantly lowered the percentage of time spent in central area **(B)**, open-arm time **(D)** and entries into open arms **(F)** relative to vehicle/vehicle injection in the two groups. The doses leading to the alterations in anxiety-like behaviors in the lesioned rats were lower than those in sham-operated rats. ^#^ *p* < 0.05, ^##^ *p* < 0.01, ^###^ *p* < 0.001 *vs*. vehicle/vehicle injection into the LHb in the same group; *p < 0.05, **p < 0.01, ***p < 0.001 *vs*. sham-operated rats; a two-way ANOVA followed by Bonferroni’s test. Data are represented as means ± SEM; *n* = 10 rats/group.

In terms of anxiety-like behaviors measured using the OFT, unilateral 6-OHDA lesions of the SNc attenuated the percentage of time spent in the central area compared to sham operations [(*S*)-AMPA: *p* < 0.05; NBQX: *p* < 0.001; unpaired Student’s t-test; Figures 4A,B]. A two-way ANOVA of (*S*)-AMPA-treated groups (lesion × drug) revealed significant main effects of lesion and drug on the percentage of time spent in the center (lesion: *F*_(1, 90)_ = 5.556, *p* < 0.05, Figure 4A; drug: *F*_(4, 90)_ = 16.414, *p* < 0.001), but without lesion × drug interaction effect (*F*_(4, 90)_ = 2.115, *p* = 0.09, Figure 4A). *Post hoc* analyses showed that the treatment with (*S*)-AMPA significantly enhanced the percentage of time spent in the central area (sham-operated rats: 0.075μg, *p* < 0.05; SNc-lesion rats: 0.0375μg, *p* < 0.001; 0.075μg, *p* < 0.001, Figure 4A).

A two-way ANOVA of NBQX-treated groups (lesion × drug) also revealed significant main effects of lesion and drug on the duration in the central area (lesion: *F*_(1, 72)_ = 52.752, *p* < 0.001, Figure 4B; drug: *F*_(3, 72)_ = 13.328, *p* < 0.001), but no interaction (*F*_(3, 72)_ = 1.644, *p* = 0.19, Figure 4B). In contrast to the effects of (*S*)-AMPA, *post hoc* analyses indicated that treatment with NBQX robustly reduced the time spent in central area in the two groups (sham-operated rats: 1.0 μg, *p* < 0.001; SNc-lesion rats: 0.5 μg, *p* < 0.01; 1.0 μg, *p* < 0.05, Figure 4B).

These results indicate that (*S*)-AMPA injections suppressed anxiety-like behaviors, while NBQX treatment augmented anxiety-like behaviors, and the dose required to produce a change in the anxiety-like phenotype in the SNc-lesion rats was lower than that in sham-operated ones (for (*S*)-AMPA: sham-operated *vs*. SNc-lesion: 0.075 μg *vs*. 0.0375 μg, Figure 4A; for NBQX: sham-operated *vs*. SNc-lesion: 1.0 μg *vs*. 0.05 μg, Figure 4B). Furthermore, the pretreatment with NBQX blocked the effect of (*S*)-AMPA on anxiety-like behavior in both sham-operated and lesioned groups (Figures 4A,B).

### Effects of SNc-Lesion and Pharmacological Manipulations of LHb AMPARs on Anxiety-like Behaviors in the EPM

In the EPM test, unilateral 6-OHDA lesion of the SNc significantly reduced the percentage of time spent in the open arms and the number of open-arm entries relative to sham-operated rats ((*S*)-AMPA: *t* = 10.254, *p* < 0.01; NBQX: *t* = 3.424, *p* < 0.001 of time spent in the open arms and (*S*)-AMPA: *t* = 2.370, *p* < 0.001; NBQX: *t* = 1.555, *p* < 0.001 of number of open-arm entries; unpaired Student’s t-test; Figures 4C–F). A two-way ANOVA of (*S*)-AMPA-treated groups (lesion × drug) revealed main effects of both lesion and drug with respect to time spent in the open arms (lesion: *F*_(1, 90)_ = 8.767, *p* < 0.05, Figure 4C; drug: *F*_(4, 90)_ = 8.497, *p* < 0.001), but no interaction (*F*_(4, 90)_ = 2.330, *p* = 0.06, Figure 4C). A two-way ANOVA of (*S*)-AMPA-treated groups (lesion × drug) also revealed significant main effects of lesion and drug with respect to open-arm entries (lesion: *F*_(1, 90)_ = 13.730, *p* < 0.05, Figure 4E; drug: *F*_(4, 90)_ = 12.163, *p* < 0.001), but no interaction (*F*_(4, 90)_ = 1.295, *p* = 0.278, Figure 4E). *Post hoc* analyses showed that (*S*)-AMPA treatment significantly raised the open-arm times and open-arm entries, indicating an increase in anxiolytic-like behavior following the injection of (*S*)-AMPA (open-arm time in sham-operated rats: 0.075 μg, *p* < 0.01; SNc-lesion rats: 0.0375 μg, *p* < 0.01; 0.075 μg, *p* < 0.01; open arm entries in sham-operated rats: 0.075, *p* < 0.01; SNc-lesion rats: 0.0375 μg, *p* < 0.001; 0.075 μg, *p* < 0.001; Figures 4C,E).

A two-way ANOVA of NBQX-treated groups (lesion by drug) revealed significant main effects of lesion and drug with respect to open arm time (lesion: *F*_(1, 72)_ = 42.376, *p* < 0.001, Figure 4D; drug: *F*_(3, 72)_ = 10.349, *p* < 0.001) but no interaction (*F*_(3, 72)_ = 0.990, *p* = 0.403, Figure 4D). A two-way ANOVA of NBQX-treated groups (lesion x drug) also revealed main effects of both lesion and drug with respect to open arm entries (lesion: *F*_(1, 72)_ = 21.674, *p* < 0.001, Figure 4F; drug: *F*_(3, 72)_ = 6.997, *p* < 0.001), but without lesion × drug interaction effect (*F*_(3, 72)_ = 0.445, *p* = 0.722, Figure 4F). In contrast to the effects of (*S*)-AMPA, *post hoc* analyses displayed that NBQX significantly decreased the percent time staying in the open arms and open arm entries of the two groups, indicating that NBQX increases anxiety-like behavior (open-arm time in sham-operated rats: 1.0 μg, *p* < 0.001; SNc-lesion rats: 0.5 μg, *p* < 0.05; 1.0 μg, *p* < 0.05; open arm entries in sham-operated rats: 1.0 μg, *p* < 0.01; SNc-lesion rats: 0.5 μg, *p* < 0.01; 1.0 μg, *p* < 0.05; Figures 4D,F).

These results again suggested that (*S*)-AMPA treatment reduced anxiety-like behaviors, while NBQX treatment induced anxiolytic-like behaviors, and the dose required to produce a change in anxiety-like behavior in the SNc-lesion groups was lower than that in sham-operated groups (for (*S*)-AMPA: sham-operated *vs*. SNc-lesion: 0.075 μg *vs*. 0.0375 μg, Figures 4C,E; for NBQX: sham-operated *vs*. SNc-lesion: 1.0 μg *vs*. 0.05 μg, Figures 4D,F). Furthermore, NBQX pretreatment blocked the effect of (*S*)-AMPA in both groups (Figures 4C–F).

### Extracellular DA and 5-HT Concentration Measurements in the BLA After SNc Lesion and Pharmacological Manipulations of LHb AMPARs

**FIGURE 5:**
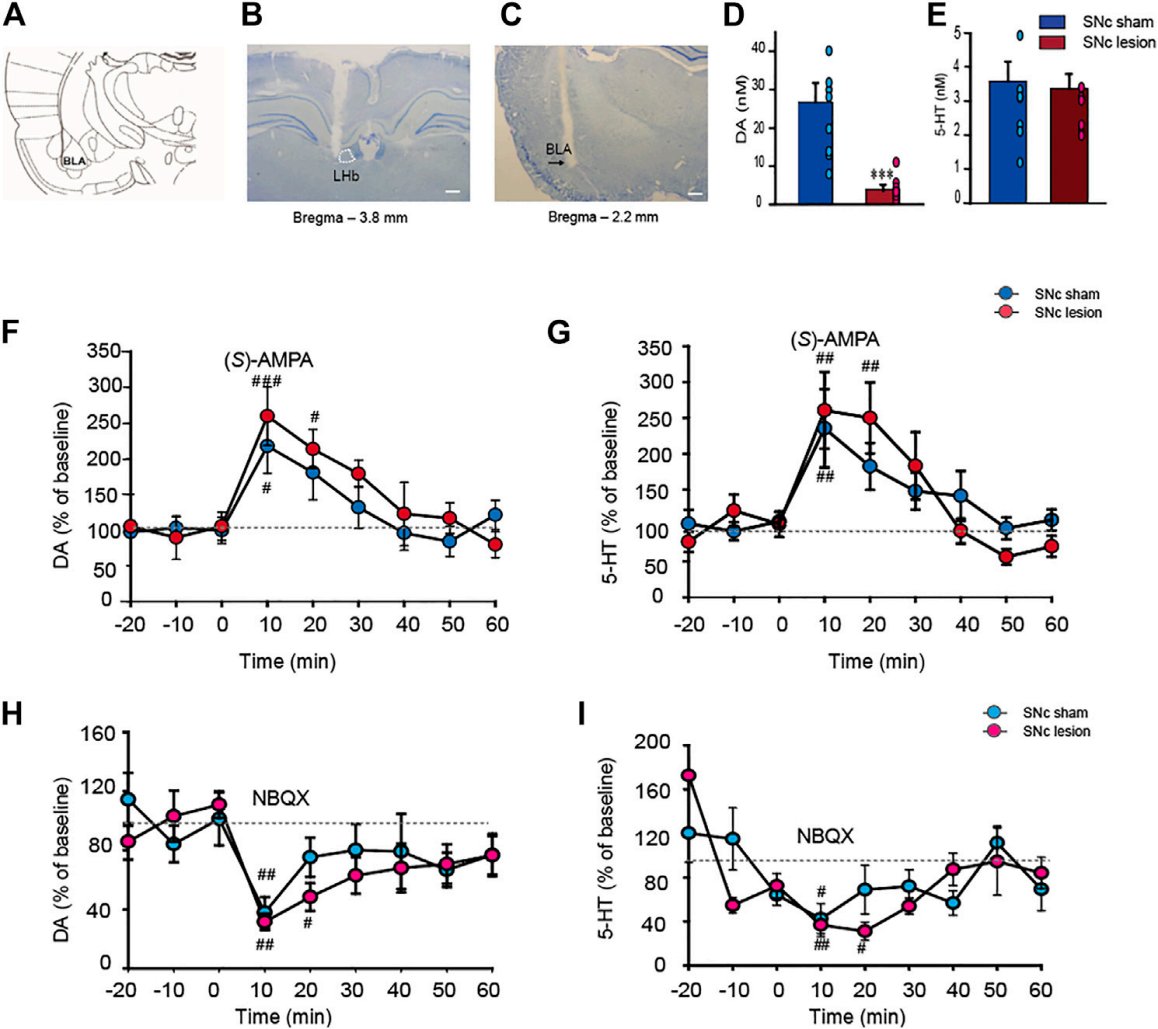
The alterations of the extracellular DA and 5-HT levels in the BLA in sham-operated and SNc-lesion rats prior to and following an intra-LHb injection of the AMPARs agonist (*S*)-AMPA or antagonist NBQX. Schematic drawing adapted from Paxinos et al. (1980). **(A)**. The microinjector tip aimed at the LHb **(B)** and photomicrographs of cresyl violet staining indicated the sites of microdialysis probes in the BLA **(C)**. Unilateral SNc lesions in rats suppressed the DA level in the ipsilateral BLA relative to sham-operated rats **(D)** but did not impact the 5-HT level [**(E)**; *n* = 9 rats/group]. Intra-LHb injection of (*S*)-AMPA (0.0375 μg/0.3 μl) raised the levels of DA and 5-HT in the BLA in both sham and SNc-lesion rats [**(F,G)**; *n* = 8 rats/group], while NBQX (0.5 μg/0.3 μl) inhibited the concentrations of DA and 5-HT in the BLA [**(H,I)**; *n* = 7 rats/group). Although (*S*)-AMPA and NBQX affected the DA and 5-HT levels in both groups, the durations of the increase or decrease were longer in the lesion group than in sham-operated rats. **p < 0.001 *vs*. sham-operated rats; ^#^ *p* < 0.05, ^##^ *p* < 0.01, ^###^ *p* < 0.001 *vs*. baseline, two-way ANOVA with repeated measures followed by Bonferroni’s test. Data are represented as means ± SEM.

SNc lesions in rats significantly suppressed the extracellular DA level in the BLA compared to sham-operated rats (-85%; *p <* 0.001, unpaired Student’s t-test; Figures 5A–D); however, the lesions did not alter the extracellular 5-HT level in the BLA (Figure 5E), indicating that lesions of the SNc specifically affect DA release in the BLA.

Intra-LHb injection of (*S*)-AMPA enhanced the extracellular DA level in the BLA compared to baseline in both sham-operated and SNc-lesion groups (Figure 5F). A two-way ANOVA (time × group) revealed a significant main effect of time (*F*_(6, 42)_ = 10.644, *p* < 0.001), but no main effect of group (*F*_(1, 42)_ = 0.807, *p* = 0.399) or time × group interaction effect (*F*_(6, 42)_ = 0.639, *p* = 0.699). (*S*)-AMPA also increased the extracellular 5-HT level in the BLA compared to baseline in the two groups (Figure 5G). A two-way ANOVA (time x group) revealed a significant main effect of time (*F*_(6, 42)_ = 13.182, *p* < 0.001), but no main effect of group (*F*_(1, 42)_ = 0.0002, *p* = 0.988) or time × group interaction effect (*F*_(6, 42)_ = 0.721, *p* = 0.635). However, the effects of (*S*)-AMPA on DA and 5-HT concentrations in the SNc-lesion group lasted much longer than that in the sham-operated group (sham-operated *vs.* SNc-lesion: DA, 10 min *vs*. 20 min; 5-HT, 10 min *vs*. 20 min; Figures 5F,G).

Conversely, an intra-LHb NBQX injection reduced the extracellular DA level in the BLA compared to baseline in both sham-operated and the SNc-lesion groups (Figure 5H). A two-way ANOVA (time × group) revealed a significant main effect of time (*F*_(6, 42)_ = 4.882, *p* < 0.001), but no main effect of group (*F*_(1, 42)_ = 1.247, *p* = 0.307) or time × group interaction effect (*F*_(6, 42)_ = 0.376, *p* = 0.889). NBQX treatment also reduced the extracellular 5-HT level in the BLA compared to baseline in both groups (Figure 5I). A two-way ANOVA (time × group) revealed a significant main effect of time (*F*_(6, 36)_ = 7.799, *p* < 0.001), but no main effect of group (*F*_(1, 36)_ = 0.134, *p* = 0.727) or time × group interaction effect (*F*_(6, 42)_ = 1.017, *p* = 0.430). Again, the pharmacological effects of NBQX on the levels of DA and 5-HT in the SNc-lesion group lasted longer than in sham-operated group (sham-operated *vs.* SNc-lesion: DA, 10 min *vs*. 20 min; 5-HT, 10 min *vs*. 20 min; Figures 5H,I).

Here, the data indicated that activating and antagonizing AMPARs in the LHb regulated DA and 5-HT release in the BLA of both groups, while DA depletion enhanced the responses of LHb neurons to AMPARs stimulation.

## Discussion

The present study examined whether AMPARs in the LHb are involved in the regulation of PD-associated anxiety-like phenotypes. We found that a unilateral lesion of the SNc led to a reduction in the number of DA neurons in the midbrain, which mimics the loss of DA neurons seen in late-stage PD patients. Furthermore, activation of AMPARs by (*S*)-AMPA induced anxiolytic-like effects and enhanced concentrations of extracellular DA and 5-HT in the BLA in both SNc-lesion and control rats; conversely, blockade of AMPARs by NBQX produced anxiety-like effects and decreased the extracellular DA and 5-HT levels in the BLA in both groups. The anxiolytic- and anxiety-like effects of activation and blockade, respectively, of AMPARs in the LHb were achieved with lower doses in the SNc lesion groups relative to the sham groups. Similarly, the drugs at lower doses also prolonged the duration of the DA and 5-HT release in the BLA in the lesioned rats.

Anxiety is a non-motor symptom commonly present in PD patients. In this study, the behavioral tests showed that unilateral SNc lesions suppressed the anxiety-like phenotypes in the OFT and EPM tests, suggesting that the depletion of DA induced anxiety-like behaviors. These results were confirmed by the previous studies from our group and supported by the publications demonstrating that rats with unilateral SNc lesions displayed augmented anxiety-like phenotypes in the EPM test, social interaction (SI) tests, and amphetamine-induced hyperlocomotion (AIH) (Hui et al., 2015; Sun et al., 2018). This study also exhibited that unilateral SNc dysfunction suppressed the DA level in the ipsilateral BLA, while the 5-HT level was unaffected, indicating that DA depletion within the cortico-limbic circuit likely plays an essential role in PD-associated anxiety.

The LHb regulates anxiety-related behaviors through its complex connection with distal regions. Based on these connections (Jhou et al., 2009; Xie et al., 2016), the LHb modulates neurotransmitter systems including DA and 5-HT, and participates in various physiological and pathophysiological processes such as anxiety, depression, reward-aversion and addiction (Nair et al., 2013; Lecca et al., 2014). Moreover, the inhibitory inputs from the LHb have been proved to involve in manipulating reward-related activities, and this function has been linked to 5-HT modulation (Teissier et al., 2015) and DA transmission (Matsumoto and Hikosaka, 2007), which can produce anxiety-like behaviors (Huang et al., 2017; Vadodaria et al., 2018). Inactivation of the LHb by GABA receptor agonists attenuates the anxiolytic-like effects as seen by an increased time staying in the open arms of the EPM test (Friedman et al., 2011; Meng et al., 2011; Gill et al., 2013), again indicating an association between the LHb and anxiety. The afferent inputs innervate the BLA neurons through the systems to maintain a balance between inhibitory and excitatory post-synaptic currents, ultimately determining the primary state of excitability of BLA efferent neurons (Rainnie et al., 1991a; Rainnie et al., 1991b). This balance plays an important role in amygdala-mediated behaviors, such as anxiety (Sajdyk and Shekhar, 1997a; Sajdyk and Shekhar, 1997b). The BLA mainly receives dopaminergic and serotonergic afferents from the VTA and raphe nuclei (Ma et al., 1991; Scibilia et al., 1992; Pinard et al., 2008). Previously research showed that the LHb plays a critical role in mediating the effects of acute and chronic nicotine by activating DA neurons (Pierucci et al., 2022) and regulating 5-HT receptors in the VTA (Bombardi et al., 2021a; Bombardi et al., 2021b). Moreover, the LHb strictly controls the activity of dopaminergic and serotonergic neurons that provide DA and 5-HT neurotransmitter to the forebrain (Lecca et al., 2014; Metzger et al., 2017), and lesions of the LHb produced anxiolytic effects in Parkinsonian rats (Du et al., 2021). However, it is unclear on how activation and blockade of AMPARs within the LHb affects the BLA in the anxiety-like behaviors. Here we observed that the intra-LHb microinjection of (S)-AMPA led to anxiolytic-like responses and enhanced the extracellular DA and 5-HT concentrations in the BLA; conversely, NBQX produced opposite effects, likely resulting from the lowered extracellular DA and 5-HT concentrations in the BLA.

Most LHb projections are glutamatergic (Geisler et al., 2007; Brinschwitz et al., 2010) and predominantly express AMPARs (Li et al., 2011; Maroteaux and Mameli, 2012; Meye et al., 2013). AMPARs consisted of four subunits (GluR1–GluR4; Traynelis et al., 2010) and can be clustered into the calcium-impermeable GluR2-containing and calcium-permeable GluR2-lacking AMPARs (Keinanen et al., 1990; Seeburg et al., 1998). AMPARs mediate rapid excitatory synaptic transmission, neuronal death, and synaptic plasticity. Our study shows that activation of LHb AMPARs produced an anxiolytic-like effect in both sham-operated rats and SNc-lesion rats. (*S*)-AMPA injected into the LHb significantly raised anxiety-like behaviors in the OFT and EPM tests, while blockade of LHb AMPARs using NBQX produced an anxiety-like behavior in both groups and suppressed the anxiety-like phenotypes in rats. Interestingly, the minimal doses inducing the effects in the SNc-lesion rats were lower than those in sham-operated rats, suggesting that the action of AMPARs in the LHb regulates anxiety-like behavior and SNc lesions enhanced the sensitivity of AMPARs.

## Conclusion

In conclusion, the intra-LHb (S)-AMPA infusion enhanced the extracellular DA and 5-HT concentrations in the BLA. In contrast, NBQX treatment decreased the release of DA and 5-HT in the BLA. In addition to the increase and decrease in anxiety-like behaviors demonstrated here by (S)-AMPA and NBQX, respectively, these findings support a role for LHb AMPARs in mediating anxiety *via* its effects on DA and 5-HT in the BLA. This study lays a foundation for further understanding the involvement of AMPARs in the LHb in the regulation of PD-related anxiety, and suggests a novel therapeutic role of AMPARs in treating PD-related anxiety.

## Compliance With Ethical Standards

The present study was performed on rats. All the experimental procedures were strictly abided by the National Institute of Health Guidelines and approved by the Animal Care and Use Committee of the Xi’an Jiao Tong University. All efforts were made to minimize the number of animals used and reduce their pain.

## Data Availability

The original contributions presented in the study are included in the article/Supplementary Materials, further inquiries can be directed to the corresponding authors.
